# Assessing the effect of scanning parameter on the size and density of pulmonary nodules: a phantom study

**DOI:** 10.1186/s12880-023-01190-4

**Published:** 2024-01-05

**Authors:** Donghua Meng, Zhen Wang, Changsen Bai, Zhaoxiang Ye, Zhipeng Gao

**Affiliations:** 1 Department of Radiology, Tianjin Medical University Cancer Institute and Hospital, National Clinical Research Center for Cancer, Key Laboratory of Cancer Prevention and Therapy, Tianjin’s Clinical Research Center for Cancer, Tianjin, China; 2 Geriatrics Department, Tianjin NanKai Hospital, Tianjin, China; 3 Department of Clinical Laboratory, Tianjin Medical University Cancer Institute and Hospital, National Clinical Research Center for Cancer, Key Laboratory of Cancer Prevention and Therapy, Tianjin’s Clinical Research Center for Cancer, Tianjin, China; 4 National Clinical Research Center for Cancer, Key Laboratory of Cancer Prevention and Therapy, Tianjin’s Clinical Research Center for Cancer, Tianjin Medical University Cancer Institute and Hospital, Huanhuxi Road, Hexi District, Tianjin, 300060 China

**Keywords:** Scanning parameter, Pulmonary nodule, Phantoms

## Abstract

**Background:**

Lung cancer remains a leading cause of death among cancer patients. Computed tomography (CT) plays a key role in lung cancer screening. Previous studies have not adequately quantified the effect of scanning protocols on the detected tumor size. The aim of this study was to assess the effect of various CT scanning parameters on tumor size and densitometry based on a phantom study and to investigate the optimal energy and mA image quality for screening assessment.

**Methods:**

We proposed a new model using the LUNGMAN N1 phantom multipurpose anthropomorphic chest phantom (diameters: 8, 10, and 12 mm; CT values: − 100, − 630, and − 800 HU) to evaluate the influence of changes in tube voltage and tube current on the size and density of pulmonary nodules. In the LUNGMAN N1 model, three types of simulated lung nodules representing solid tumors of different sizes were used. The signal-to-noise ratio (SNR) and contrast-to-noise ratio (CNR) were used to evaluate the image quality of each scanning combination. The consistency between the calculated results based on segmentation from two physicists was evaluated using the interclass correlation coefficient (ICC).

**Results:**

In terms of nodule size, the longest diameters of ground-glass nodules (GGNs) were closest to the ground truth on the images measured at 100 kVp tube voltage, and the longest diameters of solid nodules were closest to the ground truth on the images measured at 80 kVp tube voltage. In respect to density, the CT values of GGNs and solid nodules were closest to the ground truth when measured at 80 kVp and 100 kVp tube voltage, respectively. The overall agreement demonstrates that the measurements were consistent between the two physicists.

**Conclusions:**

Our proposed model demonstrated that a combination of 80 kVp and 140 mA scans was preferred for measuring the size of the solid nodules, and a combination of 100 kVp and 100 mA scans was preferred for measuring the size of the GGNs when performing lung cancer screening. The CT values at 80 kVp and 100 kVp were preferred for the measurement of GGNs and solid nodules, respectively, which were closest to the true CT values of the nodules. Therefore, the combination of scanning parameters should be selected for different types of nodules to obtain more accurate nodal data.

## Introduction

Lung cancer is one of the most common cancers and has the highest age-standardized rate of all cancers [1]. Lung cancer affects 22.5 patients per 100 000 people, and it remains a leading cause of death among cancer patients [2]. Lung cancer is generally classified as small cell lung cancer (SCLC) and non-small cell lung cancer (NSCLC). NSCLC accounts for 80–85% of lung cancers [3].

Clinical treatment strategies for lung cancer differ based on histological type, and the choice of treatment strategy has a direct impact on outcome. Two parameters commonly used in clinical practice are size and density. Selection of appropriate size and density measurements is clinically important for follow-up observation and qualitative and quantitative diagnosis of nodules [4]. Computed tomography (CT) imaging is a standard-of-care imaging modality used in the cancer treatment process that plays a key role in lung cancer screening and treatment response assessment [5]. Clinical response criteria based on CT images, such as the Response Evaluation Criteria in Solid Tumors (RECIST) and modified versions, have been developed and widely used [6, 7]. These criteria use changes in tumor size over time to monitor the tumor response. Tumor size measurement in CT images is objectively influenced by image quality. The quality of CT images results from a combination of various factors in the scanning protocol, including tube voltage, tube current, slice thickness, field of view and reconstruction methods, among which tube voltage and tube current are the most important.

The determination of optimal scanning protocols has long been investigated in order to reduce radiation dose and improve tumor recognition and discrimination. New technologies such as low-dose CT and ultra-dose CT have opened up more possibilities for lung screening [8]. Christe et al. evaluated the optimal dose level in screening chest CT and concluded that 100 kVp and 25 mAs can provide satisfactory detection of solid nodules and ground glass nodules in lung cancer [9]. Du et al. tested the screening capability for small nodules with phantom scanning and found that low-dose CT results agreed with results obtained with conventional standard chest CT [10]. Jin et al. compared lung nodule detection results under high-definition and standard-definition CT and claimed that no significant differences in image quality were noted between the two scanning protocols [11]. The studies mentioned above aim at preferred image quality for lung cancer screening but did not quantitatively calculate the influence on tumor size.

Several studies [12–14] have demonstrated that tumor density, which can be quantified by the HU value, provides extra assessment information as well. Criteria for lung nodule assessment define response patterns by changes in tumor size without specifying scanning parameters. Despite the recommendation to use standard chest CT, studies of lung nodule assessment have used a variety of scanning parameters [15]. Strauch et al. performed a meta-analysis of tumor response to cancer in dynamically enhanced CT and summarized the scanning protocols applied in the study: kVp ranged from 80 to 120 and mAs ranged from 36 to 200. Different scanning parameters result in different image quality and potentially different conclusions for response assessment [15].

The aim of this study is to evaluate the impact of CT scanning parameters (tube voltage and tube current) on tumor size and density measurement and investigate the optimal energy and mA image quality for lung nodule size and density based on a phantom study.

## Materials and methods

### Materials

**Fig. 1:**
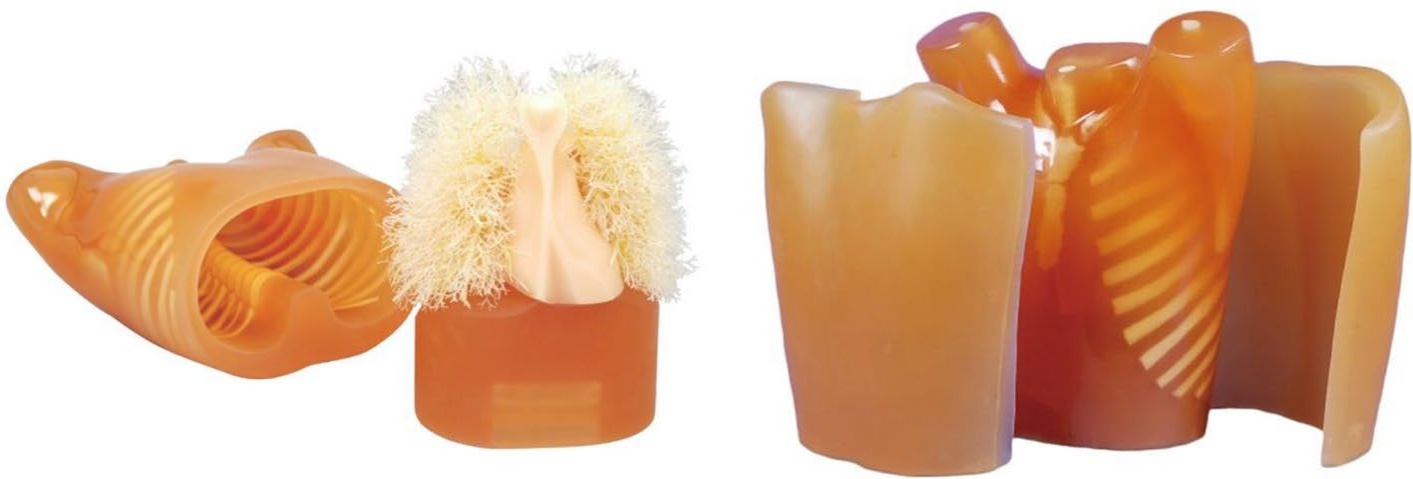
LUNGMAN phantom

We applied the LUNGMAN N1 phantom multipurpose anthropomorphic chest phantom (size, 43 × 40 × 48 cm, weight 18 kg, and chest circumference 94 cm) (Fig. 1). This chest phantom was designed by Kyoto Kagaku (Kyoto, Japan, purchased in 2021) to evaluate the influence of changes in tube voltage and tube current. The phantom is an accurate life-sized anthropomorphic model of a healthy male thorax. The rates of X-ray absorption of soft tissues and lung are similar to those of human tissues [16]. The internal structures, including the pulmonary vessels, trachea, heart, mediastinum, and some abdominal structures, are removable. These models can be used for chest X-ray and CT scan studies, as the models closely resemble the human chest [16].

In the LUNGMAN N1 model, 3 types of simulated lung nodules representing solid tumors of different sizes were used. The first nodule has a density of 100 Hounsfield units (HU) and diameters of 8, 10, and 12 mm. The second nodule has a density of − 630 HU and diameters of 8, 10, and 12 mm. The third nodule has a density of − 800 HU and diameters of 10 and 12 mm. The last two represent ground-glass nodules (GGNs), which have been validated by several studies [17, 18]. Since widely used tumor evaluation criteria, such as RECIST 1.1 [6] and iRECIST [7], consider 10 mm as the minimal measurable tumor size, we chose 8, 10 and 12 mm to simulate tumor size. A total of 8 nodules were placed in the anthropomorphic chest model. The placement positions were the upper, middle, and lower lungs.

### Image acquisition

A GE Discovery 750HD CT scanner [General Electric Co. (GE), USA] was used, and the combined scanning schemes of different tube voltages (kVp) and tube currents (mA) were adopted for the anthropomorphic chest phantom. The scanning voltage was set at 80, 100, and 120 kVp with tube currents set at 200, 140 and 100 mA, there are a total of 9 combined scanning programs to be implemented. Scanning Parameters: the pixel value was 0.78 by 0.78 mm^2^, and the slice thickness was set to 1.25 mm. The reconstructed diameter as a field of view (DFOV) was 40 cm, and the data collection diameter as a scanning field of view (SFOV) was 50 cm. The CT scanning pitch was 0.984:1.000, and the rotation time of the rack was 0.5 s. The Adaptive Statistical Iterative Reconstruction (ASiR) algorithm was used for reconstruction with a matrix of 512 × 512 [19]. The automatic exposure control (AEC) of scanner was Off during the scan. When collecting images, two screening setups, lung window reconstruction (window width of 1200 HU and window level of − 500 HU) and standard soft tissue window reconstruction (window width of 320 HU and window level of 50 HU), were applied for each acquisition sharing the same tube voltage and tube current combination (Table 1). The scanning range of the anthropomorphic chest phantom was performed from the lung apex to the lung base.

**Table 1:** Scanning parameters

Parameters	Parameter value
Devices	Discovery HD750 (HDCT)
Tube voltage (kVp)	80, 100, 120
Tube current (mA)	200, 140, 100
Slice thickness(mm)	1.25
Reconstruction series 1	Lung window (1200HU, − 500HU)
Reconstruction series 2	Soft tissue window (320HU, 50HU)

### Measurement methods

**Fig. 2:**
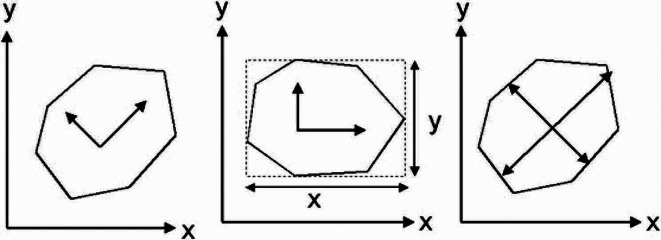
OBB diameter: the first figure indicated the direction in which the software found it, the second was to draw the box it corresponded to (the dashed one), and the third indicated the return to the original lung nodule, indicating its longest and shortest diameter positions to clearly show what the maximum and shortest diameters were

The size indicator and density of nodules needed to be collected. After acquisition and exportation, image processing was performed by 2 professional imaging physicists using 3D Slicer software. Each combination of tube voltage and tube current had two images in the lung window and soft tissue window, respectively. Segmentation for simulated solid nodules (100 HU) was performed on images reconstructed through soft tissue windows, whereas segmentation for simulated GGNs (-630 HU and − 800 HU) was performed on images reconstructed through lung windows. All the measurements were based on the segmentation of each simulated nodule. The longest diameter was used as the indicator of tumor size as outlined in the RECIST criteria and updated versions. Pulmonary nodule analysis provided quantitative information on the pulmonary nodule size through volume segmentation. The software calculated the oriented bounding box (OBB) diameters [20] in each direction and the volume of each pulmonary nodule according to the lesion segmentation (Fig. 2). The maximum diameter among all three directions was chosen to represent the largest diameter of the nodule. The region of interest (ROI) was outlined at the level with the largest diameter of the lung nodule along the edge of the nodule, and the segmentation of the outline was eroded by 2 mm (too thin a scanning layer will result in an increase in image noise, affecting the boundary of the edge of the nodule, and the erosion of 2 mm is to accurately measure the density of the nodule), and the ROIs were saved and imported in the same level of each scanning sequence to ensure that each ROI was of the same position, size, and shape. The CT and standard deviation (SD) of each ROI and the CT and SD of the image background of the same ROI at the same level were recorded separately, and the average HU of the reduced volume was calculated to indicate the density of the nodule.

We also calculated the signal-to-noise ratio (SNR) and contrast-to-noise ratio (CNR) to evaluate the image quality of each scanning combination. The SNR in a region of interest (ROI) inside the object could be described as the ratio between the mean grey value ${{\mu }}_{0}$of the ROI to the noise in that region, which was the associated standard deviation ${{\sigma }}_{0}$of the ROI’s grey values. The SNR was defined as follows:

$$ \text{S}\text{N}\text{R}= \frac{{{\mu }}_{0}}{{{\sigma }}_{0}}$$

The CNR is an important measure because it determines the detectability of defects in a volume. Using the mean grey values of background ${{\mu }}_{\text{b}}$ and object ${{\mu }}_{0}$ and calculating the noise from the standard deviation ${{\sigma }}_{\text{b}}$ of the pixel grey values in that region, CNR could be expressed as follows:

$$ \text{C}\text{N}\text{R}= \frac{\left| {{\mu }}_{0}- {{\mu }}_{\text{b}}\right|}{{{\sigma }}_{\text{b}}}$$

### Consistency analysis

The consistency between the calculated results based on segmentation from 2 physicists was evaluated using the interclass correlation coefficient (ICC) [21]. The ICC was used to assess the consistency of discrete ordinal and continuous data from multiple measures or multiple raters. 0 indicated unreliable and 1 indicated perfectly reliable. A reliability coefficient of less than 0.4 was generally considered to indicate poor reliability, and greater than 0.75 indicated good reliability. Consistency regarding both the longest tumor diameter measurement and mean HU measurement were evaluated.

### Statistical analysis

HU values for solid nodules and GGN were expressed as mean ± SD.

## Results

### Evaluation of image quality

The results for the SNR measured in the lung and soft tissue and CNR between the lung and soft tissue were shown in Table 2. The results showed that the SNR increased with the increase of tube current at the same tube voltage; the SNR increased with the rise of tube voltage at the same tube current. The CNR decreased with tube voltage at the same tube current.

**Table 2:** Image quality parameter under different image acquisition combination

Tube voltage	80 kVp			100 kVp			120 kVp		
Tube current	100 mA	140 mA	200 mA	100 mA	140 mA	200 mA	100 mA	140 mA	200 mA
SNR _soft tissue_	11.5	12.9	17.4	16.6	20.7	24.1	21.9	24.5	33.5
SNR _Lung_	43.6	46.2	56	61.1	66.2	79.2	68.4	85.7	96.8
CNR	130	125.4	127.1	118.2	117.6	112.3	101.2	104.8	104.3

### Comparison of nodule diameters measured by different scanning combinations

**Fig. 3:**
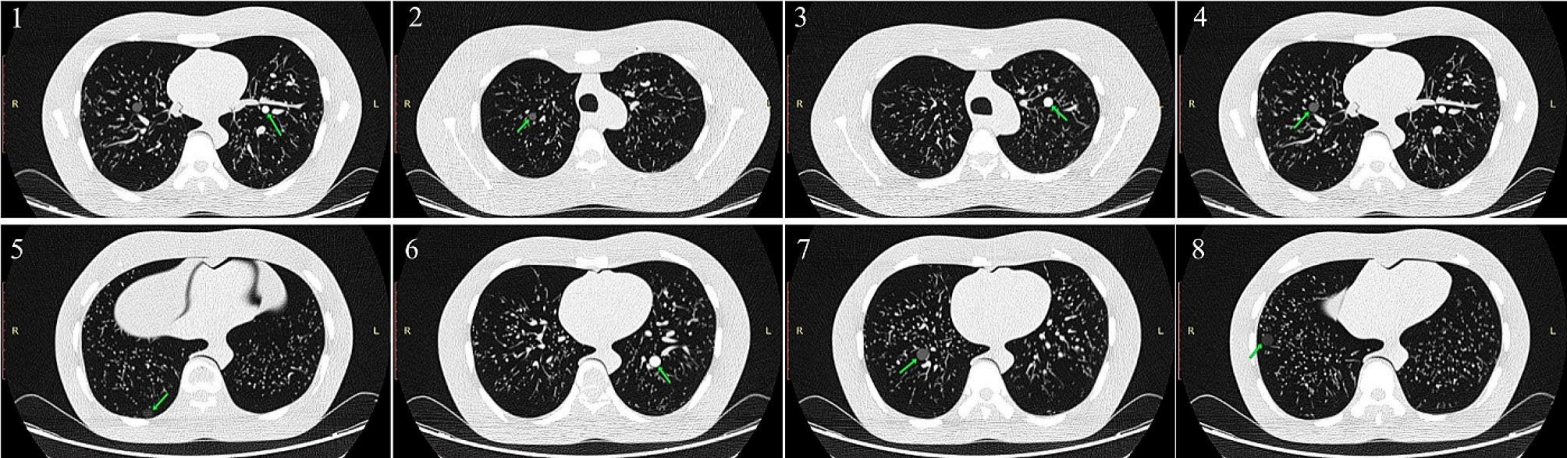
The LUNGMAN N1 model CT image. Nodule #1 is 8 mm, 100HU, nodule #2 is 8 mm, − 630HU, nodule #3 is 10 mm, 100HU, nodule #4 is 10 mm, − 630HU, nodule #5 is 10 mm, − 800HU, nodule #6 is 12 mm, 100HU, nodule #7 is 12 mm, − 630HU, nodule #8 is 12 mm, − 800HU.

In the present study, 18 sequence images (9 mediastinal window sequences, 9 lung window sequences) were obtained by 9 different scanning parameters (tube voltage, tube current). The results of the measured longest diameter of all 8 simulated nodules are shown in Table 3. The results showed that in terms of nodule size, the longest diameter of 4 out of 5 ground-glass nodules was closest to the ground truth on the images measured at a tube voltage of 100 kVp, and the longest diameter of 2 out of 3 solid nodules was closest to the ground truth on the images of the solid nodules measured at a tube voltage of 80 kVp. The CT images of the 8 nodules were shown in Fig. 3.

**Table 3:** Measurements of the longest diameter of solid nodules and GGNs under different scanning combinations

Tube voltage	Tube current	8 mm	10 mm	12 mm	8 mm	10 mm	12 mm	10 mm	12 mm
		100HU	100HU	100HU	-630HU	-630HU	-630HU	-800HU	-800HU
80 kVp		7.97	9.75	12.05	7.84	9.63	11.84	9.63	11.59
100 kVp	100 mA	7.86	10.39	12.02	**7.93**	9.67	**12.03**	9.69	11.53
120 kVp		**8.01**	9.98	12.45	8.41	9.61	11.4	9.19	11.3
80 kVp		7.81	**10**	**12.01**	8.48	9.6	11.35	**10.01**	12.16
100 kVp	140 mA	7.98	10.49	11.43	8.22	8.95	11.24	9.49	**11.93**
120 kVp		7.46	10.12	12.4	8.07	9.05	10.77	10.2	11.47
80 kVp		7.92	9.9	12.21	8.12	9.7	11.93	9.23	11.73
100 kVp	200 mA	8.09	10.34	11.66	8.41	**9.8**	11.84	9.82	12.5
120 kVp		8.43	10.02	11.56	8.1	9.46	11.56	9.56	11.27

*Note*: Bolded values indicate that the nodule size value of the measured image under this scan condition is closest to the nodule size value set by the model

### Comparison of nodule mean HU measured by different scanning combinations

Segmentation was shrunk towards the inside by 2 mm to eliminate the boundary air-pixels influencing the mean HU calculation. The mean HU and standard deviation of the shrunken volume were shown in Tables 4 and 5. The results indicated that the CT values of solid nodules were closest to the ground truth on the images of 2 of the 3 solid nodules measured at a tube voltage of 100 kVp, and the CT values of GGNs were closest to the ground truth on the images of 3 of the 5 ground-glass nodules measured at a tube voltage of 80 kVp.

**Table 4:** Measured mean HU value and standard deviation of solid nodules (Mean ± SD)

Tube voltage	Tube current	8 mm	10 mm	12 mm
		100HU	100HU	100HU
80 kVp		110.27 ± 39.53	109.50 ± 28.25	112.19 ± 22.99
100 kVp	100 mA	88.76 ± 13.97	97.61 ± 21.34	101.68 ± 18.02
120 kVp		95.23 ± 9.93	87.67 ± 15.59	93.60 ± 13.96
80 kVp		107.84 ± 26.47	106.94 ± 26.42	107.83 ± 22.63
100 kVp	140 mA	**100.00 ± 12.55**	93.16 ± 15.71	101.13 ± 14.90
120 kVp		95.48 ± 9.39	86.35 ± 14.75	94.41 ± 11.09
80 kVp		110.24 ± 16.85	**101.42 ± 23.19**	111.86 ± 17.18
100 kVp	200 mA	96.50 ± 10.69	94.97 ± 12.45	**99.83 ± 13.41**
120 kVp		95.57 ± 5.29	84.95 ± 12.86	92.82 ± 10.58

*Note*: Bolded values indicate that the nodule CT value of the measured image for that scan condition is closest to the nodule CT value set by the model

**Table 5:** Measured mean HU value and standard deviation of GGNs (Mean ± SD)

Tube voltage	Tube current	8 mm	10 mm	12 mm	10 mm	12 mm
		-630HU	-630HU	-630HU	-800HU	-800HU
80 kVp		-644.38 ± 29.60	-634.00 ± 20.83	-641.01 ± 25.95	-818.10 ± 19.64	-831.30 ± 33.15
100 kVp	100 mA	-641.31 ± 67.80	-630.29 ± 52.62	-638.17 ± 55.99	-816.07 ± 51.75	-831.74 ± 66.56
120 kVp		-640.15 ± 71.79	-637.72 ± 38.27	-643.33 ± 51.21	-820.28 ± 62.46	-828.62 ± 48.51
80 kVp		-612.93 ± 109.15	-629.60 ± 73.83	-634.90 ± 68.67	**-798.15 ± 94.14**	-834.06 ± 58.01
100 kVp	140 mA	-641.16 ± 54.04	**-629.81 ± 53.56**	-637.79 ± 50.37	-814.13 ± 62.24	-830.32 ± 49.31
120 kVp		-635.89 ± 75.87	-637.68 ± 45.04	-641.29 ± 39.36	-818.90 ± 45.88	-828.78 ± 37.22
80 kVp		**-632.58 ± 41.85**	-632.61 ± 54.81	**-634.01 ± 60.96**	-810.17 ± 83.81	-834.35 ± 52.13
100 kVp	200 mA	-640.15 ± 71.9	-634.78 ± 43.54	-638.14 ± 42.54	-817.02 ± 41.94	-830.28 ± 39.19
120 kVp		-633.01 ± 42.13	-637.44 ± 35.33	-639.29 ± 34.98	-818.53 ± 45.11	**-828.39 ± 30.34**

*Note*: Bolded values indicate that the nodule CT value of the measured image for that scan condition is closest to the nodule CT value set by the model

### Mean HU changing trend along the tube voltage

In addition to listing the calculated mean HU and SD for each scanning combination, we also calculated the HU changing trend along the tube voltage. All nodules sharing the same HU were gathered, and the mean HU was calculated regardless of their size. The results were shown in Table 6. In regard to density, the CT values of GGNs and solid nodules were closest to the ground truth when measured at 80 kVp and 100 kVp, respectively.

**Table 6:** Mean HU change based on energy

HU	80 kVp	100 kVp	120 kVp
100	108.68	97.07	91.79
-630	-633.26	-636.84	-638.06
-800	-821.02	-823.26	-823.92

### The longest tumor diameter and mean HU consistency analysis

Consistency between the results of the 2 physicists was validated using the ICC, as shown in Table 7. For the mean HU calculation, the ICC was greater than 0.99 for all scanning combinations. For the longest diameter, all ICCs were greater than 0.94. The overall consistency results showed that the measurements between the 2 physicists were consistent.

**Table 7:** Consistency results

ICC	80 kVp	100 kVp	120 kVp
Longest Diameter	0.96	0.97	0.94
Mean HU	1	1	1

## Discussion

GGNs are vague and increased shadows of lung nodules that do not cover bronchial and pulmonary vascular structures, which are often the imaging manifestations of early lung adenocarcinoma, while solid nodules are lung nodules with high density that cannot be seen through the nodules in the lung texture. With the wide clinical application of CT, the detection rate of lung nodules has been increasing, and its diagnosis and treatment have received more and more attention. Currently, the management of lung cancer screening results and the diagnostic and therapeutic evaluation of lung cancer staging are mainly based on nodule size and type.

The results of this study showed that the maximum diameter of solid nodules measured at 80 kVp and 140 mA was closer to the true size, and the maximum diameter of ground-glass nodules measured at 100 kVp and 100 mA was closer to the true size, but these differences were not statistically significant. However, for nodal CT values (HU), CT values of GGNs and solid nodules were closest to ground truth when measured at 80 kVp and 100 kVp, respectively. The focus of the study was to evaluate the effect of different tube voltage and tube current conditions on nodule size and density, and to find out the energy and mA values for obtaining the best image quality for different types of lung nodules.

The accuracy of nodule measurements is important in interpreting the possibility of whether it is a tumor, and commonly used tumor response criteria, such as RECIST 1.1 [6] and iRECIST [3], use the maximum diameter as an indicator of tumor size to monitor changes over time. On the other hand, changes in density of tumor areas before and after treatment were detected in CT images in the form of changes in HU values [16], which could enhance the injection of density changes as additional functional information into the tumor assessment criteria to further improve the assessment accuracy. This phantom study revealed that the combination of scans required for more accurate assessment of lung nodules by evaluating both aspects. It provided more accurate diagnostic information to improve the clinical management of lung nodules.

In our study, the combination of 80 kVp and 140 mA scan was preferred for solid nodule scans and 100 kVp and 100 mA scan was preferred for GGNs. The difference can be derived from each component during image acquisition. In addition to tube current and tube voltage differences, variations in field of view and slice thickness can affect image quality and subsequent measurements. Differences in scanners can have a significant impact, as vendors are equipped with different technologies based on mechanical and reconstruction methods to obtain good image quality. In clinical applications, it is important to consider how emerging reconstruction methods (e.g., ASiR) compare with classical filtered projection back (FPB) in terms of image quality [22, 23], but the latest deep learning image reconstruction (DLIR) techniques in CT will gradually be applied in clinical practice, providing more choices of “optimal” scanning parameters. Jiang et al. demonstrated that DLIR reduced image noise, improved nodule detection and measurement accuracy on ultra-low-dose chest CT images compared to adaptive statistical iterative reconstruction-V [24].

The present study has the following limitations. First, in this study, body models were used for experimental purposes. So the conclusion needs to be verified by further clinical applications. The body model used in this study was based on 70 kg adult males. Therefore, further studies are needed to determine whether this body model is suitable for other body sizes and body types. Second, in this study, only one CT scanner was used to acquire images. Therefore, further validation is needed to determine the feasibility of other types of CT scanners as well as other computer-aided design software. Third, in this study, the diameters of the simulated pulmonary nodules were 8, 10, and 12 mm. Although these diameters simulated CT Hounsfield unit values of − 100 HU and − 800 HU (tube voltage: 120 kVp), these diameters do not fully simulated lung nodules encountered in clinical work, considering the significant differences in size, shape, CT attenuation values, and other aspects of the lung nodules [25, 26]. Therefore, further in-depth studies are needed to validate the findings of this study. Finally, only three energies were included in the study. In the standard scanning procedure, the images contained both photoelectric scattering and Compton scattering. More energies with separation between these two phenomena will improve the image quality and are therefore desired for optimizing the images used for tumor response assessment. The dual energy technique is suitable for this situation and we will follow this direction.

## Conclusion

A LUNGMAN N1 body model multifunctional anthropomorphic chest model with two types of artificial lung nodules (diameters: 8, 10, and 12 mm; CT values: − 100, − 630, and − 800 HU) was used in the present study and demonstrated that a combination of 80 kVp and 140 mA scans was preferred for measuring the size of the solid nodules, and a combination of 100 kVp and 100 mA scans was preferred for measuring the size of the GGNs. However, when measuring the CT values of GGNs and solid nodules, 80 kVp and 100 kVp were preferred, respectively, and the CT values were closest to the true CT values of the nodules. Therefore, the combination of scanning parameters should be selected for different types of nodules to obtain more accurate nodal data.

## Data Availability

Datasets used and/or analyzed during the current study are available from the corresponding author upon reasonable request.
